# Methylenetetrahydrofolate reductase (*MTHFR*) gene C677T (rs1801133) polymorphism and risk of alcohol dependence: a meta-analysis

**DOI:** 10.3934/Neuroscience.2021011

**Published:** 2021-01-27

**Authors:** Vandana Rai, Pradeep Kumar

**Affiliations:** Human Molecular Genetics Laboratory, Department of Biotechnology, VBS Purvanchal University, Jaunpur-222 003, UP, India

**Keywords:** alcohol dependence, AD, *MTHFR*, C677T, polymorphism, homocysteine, meta-analysis

## Abstract

Alcohol dependence is a complex neuropsychiatric disorder. Numerous studies investigated association between *MTHFR* gene C677T (rs1801133) polymorphism and alcohol dependence (AD), but the results of this association remain conflicting. Accordingly, authors conducted a meta-analysis to further investigate such an association. PubMed, Elsevier Science Direct and Springer Link databases were searched for studies on the association between the *MTHFR*C677T polymorphism and AD. Pooled odds ratio (OR) with 95% confidence interval (CI) was calculated using the fixed- or random-effects model. Statistical analysis was performed with the software program MetaAnayst and MIX.A total of 11 articles were identified through a search of electronic databases, up to February 28, 2020. The results of the present meta-analysis did not show any association between MTHFR C677T polymorphisms and AD risk (for T vs. C: OR = 1.04, 95% CI = 0.88–1.24; CT vs. CC: OR = 1.02, 95% CI = 0.62–1.68; for TT+CT vs. CC: OR = 1.10, 95% CI = 0.94–1.29; for TT vs. CC: OR = 1.01, 95% CI = 0.66–1.51; for TT vs. CT+CC: OR = 0.97, 95% CI = 0.66–1.40). Results of subgroup analysis showed no significant association between *MTHFR* C677T polymorphism with AD in Asian as well as in Caucasian population. In conclusion, C677T polymorphism is not a risk factor for alcohol dependence.

## 1. Introduction

Alcohol dependence (AD) or Alcoholism, also regarded as alcohol use disorder (AUD), is a complex and relapsing neuropsychiatric disorder [1],[2]. World Health Organization (WHO) reported that approximately 140 million individuals addicted to alcohol globally, resulting in to 2.5 million death each year [3]. AD is regarded as a “reward deficiency syndrome” that intemperately affects public health [4],[5]. It has been found to be influenced by both genetic and environmental factors [6],[7]. Exact patho-physiological and molecular mechanism of AD is not known yet. However, molecular genetic studies support that multiple genes determine an individual's predisposition to AD [8]. Heritability of AD likely plays an important role in its development and is determined to be moderate to high [9],[10]. It was reported frequently that alcohol consumption increased homocysteine (Hcy) concentration i.e hyperhomocysteinaemia [11]. However, inconsistent results of the combined effect of both positive and negative association have been reported between alcohol intake and Hcy [12]. Hyperhomocystenemia is already reported as risk factor for several diseases or disorders including neural tube defects, Alzheimer disease, schizophrenia, pregnancy complications, cardiovascular diseases, noninsulin dependent diabetes and end-stage renal disease as evidenced from several studies [13].

Homocysteine is a sulfur containing amino acid, several genetic and environmental risk factor are reported for higher plasma concentration of homocysteine [14]. Homocysteine (Hcy) is synthesized in methionine and folate cycle by demethylation of methionine. 5,10-methylenetetrahydrofolate reductase (*MTHFR*) enzyme of folate cycle plays an important role in homocysteine metabolism. *MTHFR* gene is present on chromosome 1p36.3. Numerous single nucleotide polymorphisms (SNP) are known in *MTHFR* gene like C677T and A1298C etc [15],[16]. The most clinically important and studies polymorphism is C677T (rs1801133), in which cytosine (C) is substituted with thymine (T) at 677 nucleotide position and consequently alanine is replaced by valine in MTHFR enzyme (Ala 222 Val) [17],[18]. The variant MTHFR enzyme is thermolabile with reduced activity (~70%) and it increased the plasma homocysteine concentrations [15] (Frosst et al., 1995). Globally, frequency of mutant T allele varies greatly [19]–[23]. Yadav et al. [23] have conducted a comprehensive C667T polymorphism study and reported the highest frequency in European populations ranging from 24.1% to 64.3% and, lowest frequency from African population. Several studies revealed association of *MTHFR* gene C677T polymorphism with AD. However, findings showed inconsistent results [24]–[26]. To derive a more precise estimation of the relationship, authors have performed a meta-analysis.

## 2. Methods

Present meta-analysis is carried out according to MOOSE (Meta-analysis of observational studies in epidemiology) guidelines.

### 2.1. Retrieval strategy and selection criteria

Articles were retrieved through Pubmed, Google scholar, Springer Link, and Science Direct databases up to February 28, 2020, using following key words: “Methylenetetrahydrofolate reductase” or “*MTHFR*” or “C677T” or “rs1801133” or “polymorphism” and “Alcohol dependence” or “Alcoholism” or “AD” or “Addiction”.

### 2.2. Inclusion and exclusion criteria

Inclusion criteria were following: (1) *MTHFR* C677T polymorphism and alcohol dependence association was investigated in the study, (2) *MTHFR* C677T genotype/allele numbers in alcohol dependence cases and controls were given in the study, (3) sufficient information for calculating the odds ratio (OR) with 95% confidence interval (CI) and (4) Articles published in English language were only considered. Major reasons for studies exclusion were as follows: (1) no alcohol dependence cases analyzed, (2) the C677T polymorphism details information missing, and (3) duplicate article.

### 2.3. Data extraction

Name of first author, country name, number of cases and controls, number of genotypes in cases and controls and journal name with full reference from each article were extracted.

### 2.4. Statistical analysis

All analysis were done according to the method of Rai et al. [27]. Odds ratio (ORs) with 95% confidence intervals (CIs) were calculated using fixed effect and random effect models [28],[29]. A five genetic models viz. allele contrast, co-dominant, homozygote, dominant and recessive models were calculated. Heterogeneity was investigated and quantified by I^2^ statistic [30]. Chi-squared analysis was used to determine whether the genotype distribution of control group was in Hardy–Weinberg equilibrium or not. Subgroup analysis was conducted by ethnicity. In included articles, case samples were not categorized on the basis of gender, so the subgroup analysis based on gender did not performed in present meta-analysis. Publication bias was investigated by Egger's regression intercept test [31]. P value <0.05 was considered statistically significant. All calculations were done by softwares MIX version 1.7 [32] and MetaAnalyst [33] program.

## 3. Results

### 3.1. Eligible studies

**Figure 1.**
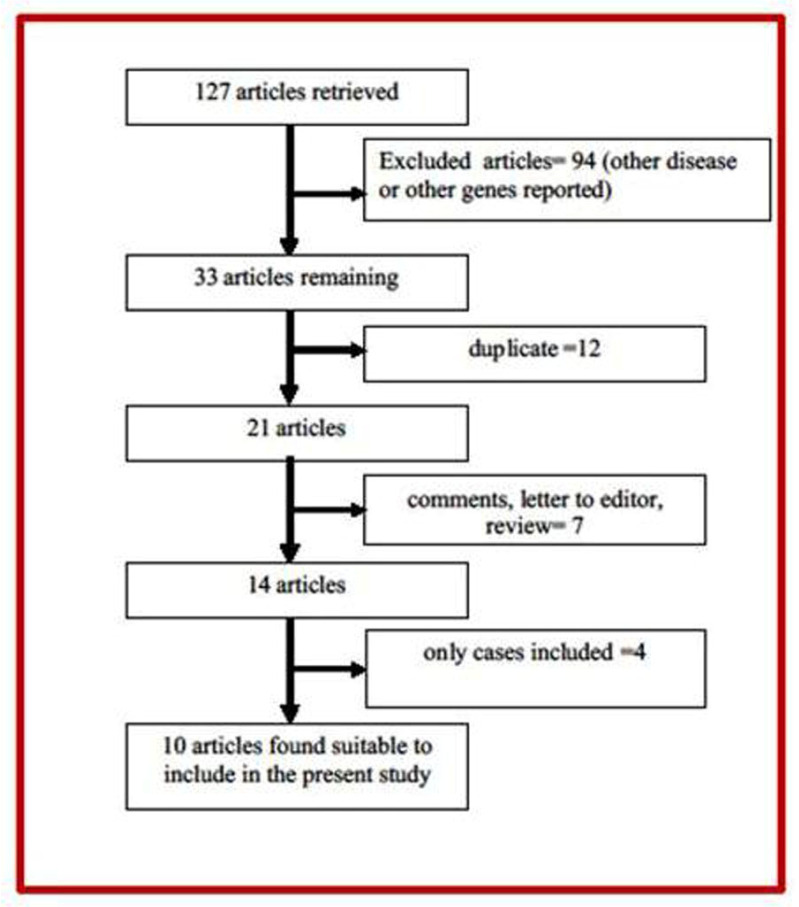
Flow diagram of study search and selection process.

Selection of studies is given in fow diagram (Figure 1). Following the exclusion criteria, 10 individual case-control studies with a total of 1676 cases and 1594 controls were included into this meta-analysis [24]–[26],[34]–[40]. One author [38] reported their data in to two categories, we included both set of data as different studies. Hence, total number of included studies in present meta-analysis is eleven (Table 1).

**Table 1.** Details of included eleven studies in the present meta-analysis.

Study	Ethnicity	Control	Case	Case Genotype			Control Genotype			HWE p-value of controls
				CC	CT	TT	CC	CT	TT	
Bonsch, 2006	Caucasian	115	134	64	56	14	60	41	14	0.10
Lutz, 2006	Caucasian	102	221	95	94	32	53	41	8	0.98
Lutz, 2007	Caucasian	101	142	65	58	19	53	40	8	0.90
Saffroy, 2008	Caucasian	93	242	108	113	21	35	41	17	0.41
Benyamina, 2009	Caucasian	93	120	56	53	11	35	41	17	0.41
Fabris, 2009	Caucasian	236	63	17	35	11	69	113	54	0.55
Shin, 2010	Asian	232	68	11	39	18	42	129	61	0.07
Supic, 2010, Heavy Alcoholic	Caucasian	57	32	13	9	10	27	24	6	0.84
Supic, 2010, Non heavy Alcoholic	Caucasian	105	64	37	23	4	53	42	10	0.69
Singh, 2014	Asian	313	139	91	44	4	228	78	7	0.91
Singh, 2015	Asian	147	451	312	125	14	107	35	5	0.32

### 3.2. Summary statistics

**Figure 2.**
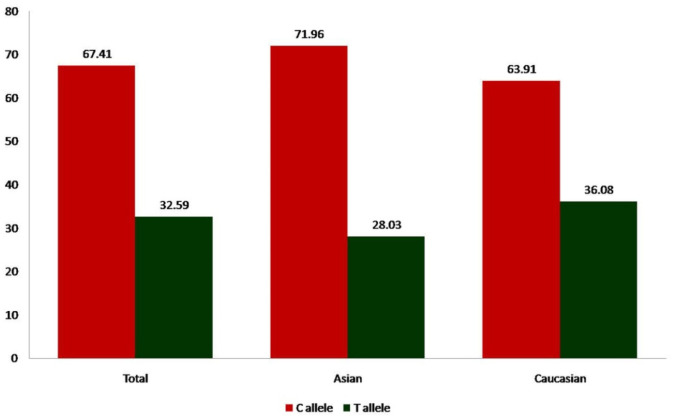
Bar diagram showing percentage of C and T allele frequencies in control group of total 11 studies, 3 Asian studies and 8 Caucasian studies.

Overall, eleven studies provided 1676/1594 cases/controls for *MTHFR* C677T polymorphism. The prevalence of C and T alleles in AD cases was 71.22% and 28.79% respectively. The percentage frequency of TT genotype among cases and controls was 9.43% and 12.98%, respectively whereas prevalence of CT heterozygote among AD cases was 38.72% and 39.21% in controls. The prevalence of CC homozygote among AD cases and controls was 51.85% and 47.80%, respectively. Genotypes were in Hardy-Weinberg equilibrium in all controls. In control group the percentage of C and T allele frequencies was 67.41% and 32.59% respectively (Figure 2).

### 3.3. Meta-analysis

**Figure 3.**
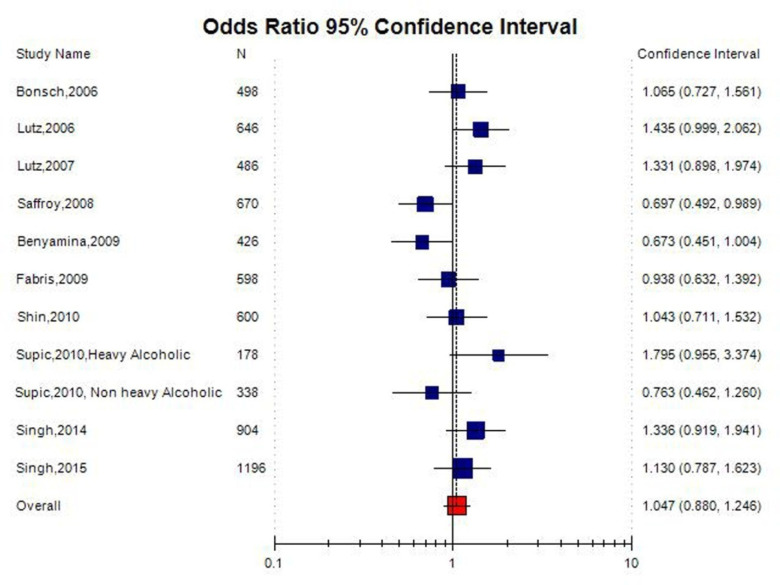
Random effect Forest plot of allele contrast model (T vs. C) of total 11 studies of MTHFR gene C677T polymorphism.

No significant association was observed between the *MTHFR* C677T polymorphism and the susceptibility to AD in all the genetic models using random effect model (for T vs. C (allele contrast): OR = 1.04, 95% CI = 0.88–1.24; CT vs. CC (co-dominant): OR = 1.02, 95% CI = 0.62–1.68; for TT+CT vs. CC (dominant): OR = 1.10, 95% CI = 0.94–1.29; for TT vs. CC (homozygote): OR = 1.01, 95% CI = 0.66–1.51; for TT vs. CT + CC (recessive): OR = 0.97, 95% CI = 0.66–1.40) (Table 2; Figures 3).

A true heterogeneity existed between studies for allele contrast (P_heterogeneity_ = 0.02, Q = 20.64, I^2^ = 51.56%, t^2^ = 0.04, z = 0.69), co-dominant genotype (P_heterogeneity_ < 0.0001, Q = 86.64, I^2^ = 88.46%, t^2^ = 0.61, z = 4.29), homozygote genotype (P_heterogeneity_ = 0.02, Q = 20.93, I^2^ = 52.24%, t^2^ = 0.24, z = 0.1), and recessive genotype (P_heterogeneity_ = 0.02, Q = 21.00, I^2^ = 52.40%, t^2^ = 0.20, z = 0.47) comparisons. The “I^2^” value of more than 50% shows high level of true heterogeneity.

**Table 2.** Summary estimates for the odds ratio (OR) of MTHFR C677T in various allele/genotype contrasts, the significance level (p value) of heterogeneity test (Q test), and the I^2^ metric and publication bias p-value (Egger Test).

Genetic Models	Fixed effect OR (95% CI), p	Random effect OR (95% CI), p	Heterogeneity p-value (Q test)	I^2^ (%)	Publication Bias (p of Egger's test)
Allele Contrast (T vs. C)	1.04 (0.92–1.17), 0.48	1.04 (0.88–1.24), 0.60	0.02	51.56	0.62
Dominant (TT+CT vs. CC)	1.41 (1.20–1.65), <0.0001	1.02 (0.62–1.68), 0.92	<0.0001	88.46	0.06
Homozygote (TT vs. CC)	0.98 (0.75–1.29), 0.92	1.01 (0.66–1.51), 0.97	0.02	52.24	0.26
Co-dominant (CT vs. CC)	1.10 (0.94–1.29), 0.21	1.10 (0.94–1.29), 0.21	0.43	0	0.48
Recessive (CC+CT vs. TT)	0.94 (0.73–1.20), 0.63	0.97 (0.66–1.40), 0.86	0.02	52.4	0.28

### 3.4. Subgroup analysis

**Figure 4.**
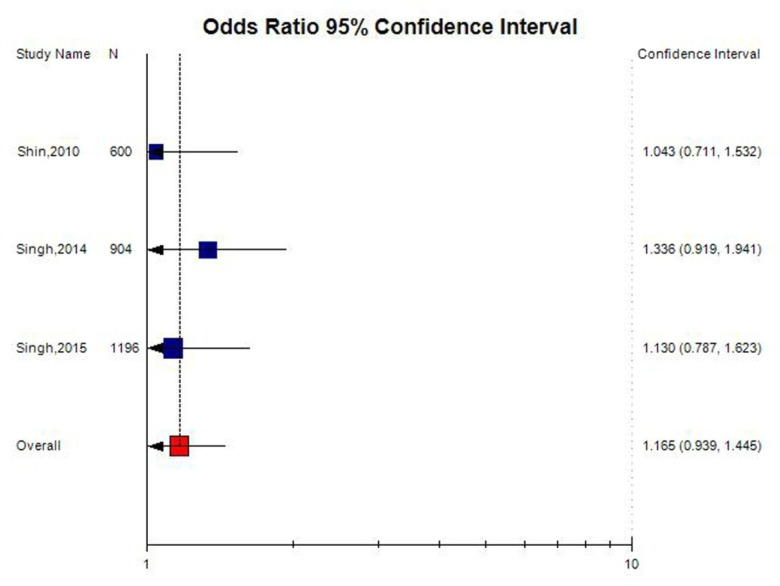
Random effect Forest plot of allele contrast model (T vs. C) of total 3 Asian studies of MTHFR gene C677T polymorphism.

**Figure 5.**
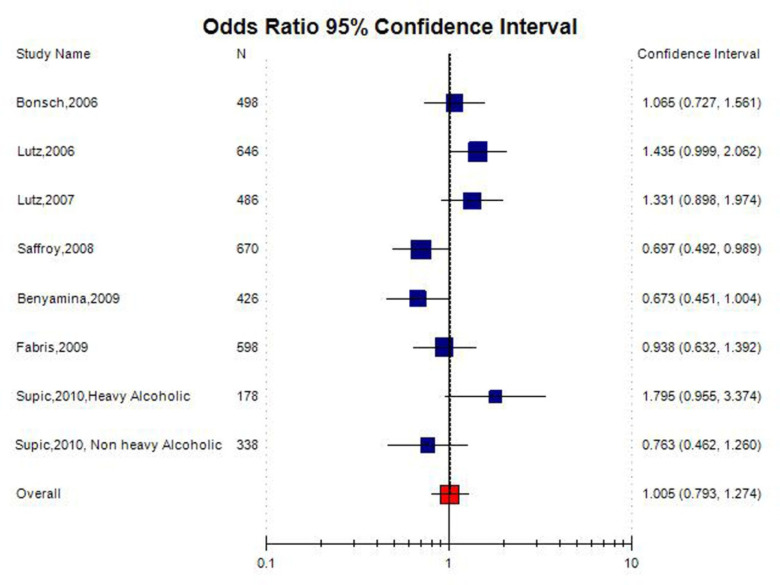
Random effect Forest plot of allele contrast model (A vs. G) of total 8 Caucasian studies of MTHFR gene C677T polymorphism.

Out of 11 studies included in the present meta-analysis, 3 studies were carried out in Asian countries, and 8 studies were carried out on Caucasian (Table 1). The subgroup analysis by ethnicity did not reveal any significant association between *MTHFR* C677T polymorphism and AD in Asian population (T vs. C: OR = 1.16; 95% CI = 0.93–1.44; p = 0.17; I^2^ = 3.1%; P_heterogeneity_ = 0.65; TT vs. CC: OR = 1.16; 95% CI = 0.62–2.02; p = 0.69; I^2^ = 3.1%; P_heterogeneity_ = 0.89; and TT+CT vs. CC: OR = 1.26; 95% CI = 0.96–1.67; p = 0.09; I^2^ = 3.1%; P_heterogeneity_ = 0.81); and Caucasian population (T vs. C: OR = 0.99; 95% CI = 0.86–1.14; p = 0.93; I^2^ = 61.75%; P_heterogeneity_ = 0.01; TT vs. CC: OR= 0.95; 95% CI = 0.70–1.29; p = 0.75; I^2^ = 65.63%; P_heterogeneity_ = 0.004; and TT+CT vs. CC: OR = 1.03; 95% CI = 0.85–1.25; p = 0.75; I^2^ = 13.93%; P_heterogeneity_ = 0.32) (Figures 4 and 5).

### 3.5. Publication bias

**Figure 6.**
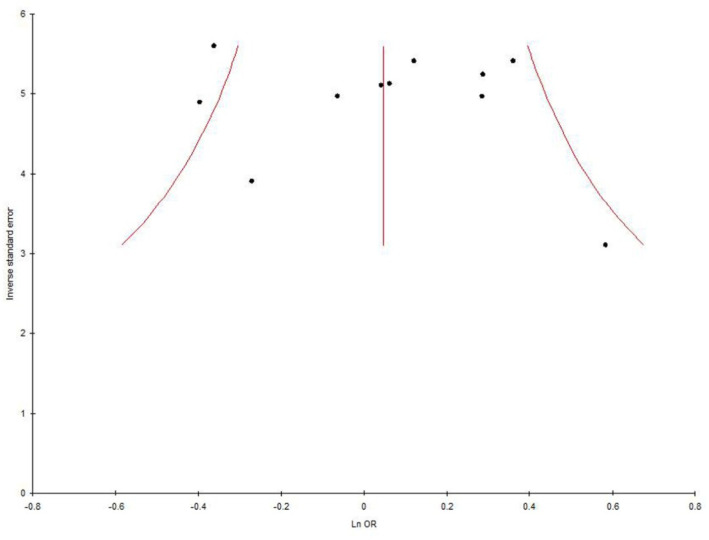
Funnel plot- Precision by log odds ratio for allele contrast model (T vs. C) of total 11 studies of MTHFR gene C677T polymorphism.

Symmetrical shape of Funnel plots' revealed absence of publication bias. P values of Egger's test were more than 0.05, also provided statistical evidence for the funnel plots' symmetry (p = 0.62 for T vs. C; p = 0.48 for TT vs. CC; p = 0.26 for CT vs. CC; p = 0.48 for TT+AC vs. CC; p = 0.28 for TT vs. CT+CC) (Table 2; Figure 6).

## 4. Discussion and conclusions

In vivo and *in vitro* studies has demonstrated that homocysteine has neurotoxic effects especially on dopamine neurons of reward pathway [11]. In addition, hyperhomocysteinemia is also reported in AD [11],[41]. In MTHFR gene several polymorphisms are reported but according to deficit hypothesis of addiction, only C677T polymorphism-dependent alteration of the reward system possibly leads to alcohol addiction. Further, homovanillic acid (HVA) is a potential indicator of central dopaminergic neuronal activity [42] and experimentally, it was demonstrated that higher concentration of homocysteine lowers the level of HVA in rat striatum region [43]. On the basis of 11 studies providing data on *MTHFR* C677T genotype and AD risk in two ethnic populations, including over 3,205 subjects, our meta-analysis provides an evidence that TT and CT genotypes or T allele are not associated with AD risk. Hence the present meta-analysis indicated that C677T is not a risk factor of AD.

Meta-analysis is a statistical tool to combine the information of independent case-control studies with similar target [44]. Several meta-analysis are published, which evaluated effects of folate pathway genes polymorphisms in susceptibility of diseases/disorders- cleft lip and palate [45], down syndrome [46]–[48], male infertility [49], bipolar disorder [50], schizophrenia [51],[52], depression [53], obsessive compulsive disorder [54], hyperurecemia [55], epilepsy [56], Alzheimers disease [57], esophageal cancer [58], and ovary cancer [59].

Several limitations that should be acknowledged like (i) calculated crude Odds ratio, (ii) included the less number of available studies (10 studies) and the limited sample size of each included study, (iii) observed higher between study heterogeneity, (iv) considered only one gene polymorphism and (v) not considered other confounding factors like diet, gender etc. In addition to limitations, current meta-analysis has several strength also such as—higher study power and larger sample size in comparison to individual case control studies, and absence of publication bias etc.

In conclusion, pooled analysis of data from 11 separate articles indicates that the *MTHFR* 677TT genotype is not a risk factor for AD. The results of present meta-analysis should be interpreted with certain cautions due to presence of higher heterogeneity and small number of included studies. Future large-scale, population-based association studies from different regions of the world are required to investigate potential gene-gene and gene-environment interactions involving the *MTHFR* C677T polymorphism in determining AD risk.
